# SUCCESSFUL AGING AMONG OLDER PERSONS IN VIET NAM

**DOI:** 10.1093/geroni/igad104.1160

**Published:** 2023-12-21

**Authors:** Yasuhiko Saito, Nguyen Vu, Linh Dang

**Affiliations:** Nihon University, Chiyoda-ku, Tokyo, Japan; Institute of Population, Health and Development, Hanoi, Ha Noi, Vietnam; Institute of Population, Health and Development, Hanoi, Ha Noi, Vietnam

## Abstract

The present study aims to explore the prevalence and correlates of successful ageing among Vietnamese older persons. Data used for this study is from the baseline survey of the Longitudinal Study of Ageing and Health in Viet Nam (LSAHV). LSAHV is a nationally representative survey of older persons aged 60 and above in 2018-19 with 6,050 respondents. Face-to-face interviews were conducted using structured questionnaires and tablets. Rowe and Kahn’s definition was used to study successful ageing among older persons in Viet Nam. Study sample size was 4,370 older persons. Logistic regression was employed to examine the association of sociodemographic and lifestyle factors with successful aging among older persons. The prevalence of Vietnamese older persons meeting criteria of successful ageing was 16.2% at the time of baseline survey. The percentage of older persons reporting no major diseases, no disability, high cognitive function, high physical function, and engagement with life was 35.0%, 88.5%, 55.1%, 77.9%, and 79.7%, respectively. Logistic regression analysis showed that older persons who were younger, male, having higher education level, and often doing physical exercise were likely to be successful agers. The prevalence of successful ageing was quite low among Vietnamese older people. More health interventions, successful ageing guidelines, and health care services should be provided for older persons to improve successful ageing status. It is necessary to study successful ageing to improve well-being of older persons becaise Viet Nam is currently experiencing a rapid population ageing.

